# Metastase axillaire d'un carcinome papillaire de la thyroïde: à propos d'un cas

**DOI:** 10.11604/pamj.2013.16.12.1478

**Published:** 2013-09-12

**Authors:** Rhizlane El Khiati, Laila Ouaissi, Sami Rouadi, Redallah Abada, Mohamed Mahtar, Mohamed Roubal, Abdellah Janah, Mustapha Essaadi, Fatmi Kadiri

**Affiliations:** 1 Service ORL Hôpital 20 Aout Casablanca, Morocco

**Keywords:** Carcinome papillaire, thyroïde, métastase axillaire, pronostic, papillary carcinoma, thyroid, axillary metastasis, prognosis

## Abstract

Les métastases axillaires dans le cadre d'un cancer de la thyroïde sont extrêmement rares. Plusieurs hypothèses expliquent ce drainage. Nous rapportons le cas d'un patient de 78 ans ayant pour antécédents chirurgicaux une lobo-isthmectomie en 1987, puis une totalisation chirurgicale en 1997 non documentés, admis en septembre 2008 pour exploration d'une masse latéro-cervicale gauche. Le reste de l'examen clinique révèle la présence de multiples adénopathies axillaires bilatérales. La biopsie exérèse d'une adénopathie axillaire droite (côté controlatéral) retrouve une métastase ganglionnaire d'un carcinome papillaire de la thyroïde avec effraction capsulaire. La tomodensitométrie cervico-thoracique note la présence d'un processus tumoral latéro-cervical gauche, un lobe thyroïdien droit siège de multiples nodules hypodenses, des adénopathies cervicales et axillaires et des lésions suspectes au niveau du parenchyme pulmonaire. Une thyroïdectomie totale avec curage ganglionnaire cervical est décidée, complétée par une ablation des ganglions axillaires macroscopiquement atteints. Des cures d'iode radioactif (IRA-thérapie) sont indiquées. Bien qu'exceptionnelle, la présence de métastases axillaires d'un carcinome thyroïdien est de pronostic péjoratif. On se demande alors si ces patients ne nécessitent pas une prise en charge particulière. Une réflexion à une stratégie thérapeutique est donc nécessaire.

## Introduction

Le carcinome papillaire de la thyroïde est le type histologique le plus fréquemment rencontré dans les cancers de la thyroïde. Il représente 60% des cancers thyroïdiens. Leur diffusion est essentiellement lymphatique sous forme de métastases ganglionnaires, cervicales et sus claviculaires [1–2]. Les métastases axillaires sont extrêmement rares. De rares cas ont été décrits dans la littérature [3, 4]. Nous rapportons le cas d'un carcinome papillaire avec métastases axillaires bilatérales, et discuterons les implications cliniques et thérapeutiques.

## Patient et observation

**Figure 1:**
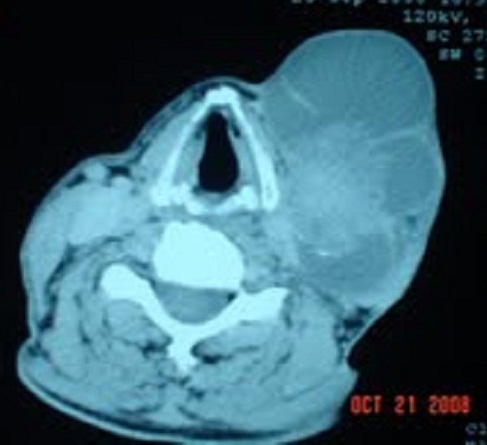
Tomodensitométrie cervicale: Processus solido-kystique latéro-cervical gauche, dont la composante charnue est hétérodense, irrégulière, siège de calcifications mesurant 92x53x78 mm

**Figure 2:**
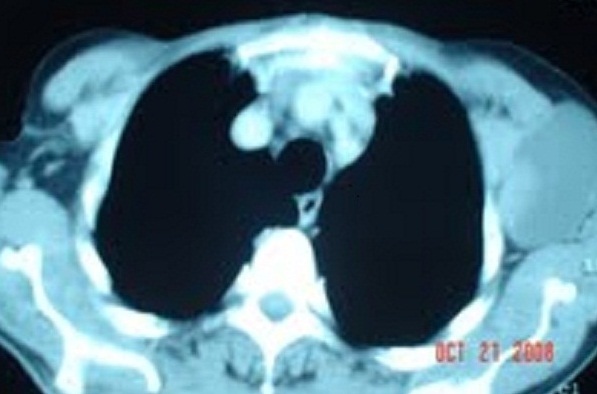
Tomodensitométrie thoracique: Volumineuse adénopathie axillaire gauche largement nécrosée

**Figure 3:**
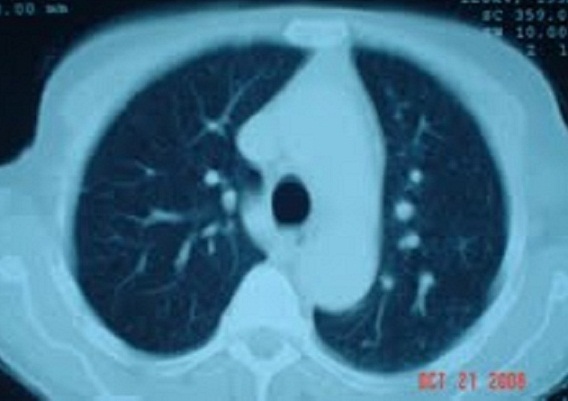
Tomodensitométrie thoracique: Micronodules parenchymateux pulmonaires

Monsieur E L, âgé de 78 ans, ayant pour antécédents chirurgicaux une lobo-isthmectomie en 1987, puis une totalisation chirurgicale en 1997 non documentés, admis en septembre 2008 pour exploration d'une masse latéro-cervicale gauche, ferme et mobile par rapport aux plans profond et superficiel, de 8x9 cm. Le reste de l'examen clinique révèle la présence de multiples adénopathies axillaires bilatérales, fermes et mobiles dont la plus volumineuse mesure 9 cm de grand axe. La biopsie exérèse d'une adénopathie axillaire droite (côté controlatéral) retrouve une métastase ganglionnaire d'un carcinome papillaire de la thyroïde avec effraction capsulaire. La tomodensitométrie cervico-thoracique note la présence d'un processus tumoral latéro-cervical gauche, largement nécrosé de 9x8x6cm (Figure 1), un lobe thyroïdien droit siège de multiples nodules hypodenses, des adénopathies cervicales et axillaires (Figure 2), et des lésions suspectes au niveau du parenchyme pulmonaire (Figure 3). Une thyroïdectomie totale avec curage ganglionnaire cervical est décidée, complétée par à une ablation des ganglions axillaires macroscopiquement atteints. Des cures d'iode radioactif (IRA-thérapie) sont indiquées.

## Discussion

Les métastases axillaires dans le cadre d'un cancer de la thyroïde sont extrêmement rares. Plusieurs hypothèses expliquent ce drainage. Rouvière [5] rapporte une communication entre les systèmes lymphatiques cervical et axillaire. Le flux physiologique est centripète jusqu’à la jonction jugulo-sous-clavière, puis emprunte cette communication pour rejoindre les lymphatiques axillaires. Une autre hypothèse peut expliquer les métastases axillaires en matière de cancer de la thyroïde: le drainage rétrograde [6]. Une compression du confluent jugulo-sous-clavier bloque le flux lymphatique. La lymphe empreinte alors la chaîne cervicale transverse pour atteindre les ganglions axillaires. Les obstacles pouvant entraîner cette compression peuvent être des métastases ganglionnaires cervicales, de la fibrose post-chirurgicale ou post-radiothérapie. Enfin, la métastase axillaire peut apparaître dans le cadre d'une diffusion hématogène et non lymphatique [6, 7]. Les cas rapportés dans la littérature sont au nombre de 10, tout type histologique confondu [8, 9]. Six d'entre eux présentaient un carcinome papillaire. Le sexe ratio est 1. L'age moyen est de 50,9 ans avec des extrêmes de 21 ans et 75 ans. Notre patient a 78 ans.

L'apparition des métastases axillaires est concomitante au cancer de la thyroïde dans 5 cas. Dans les autres cas, le délai d'apparition est compris entre 7 mois et 41 ans. Dans notre cas, la métastase axillaire est apparue 21 ans plus tard. Les métastases à distance sont rapportées dans 8 cas sur 10.

Les types histologiques étaient différents: 7 papillaires, 1 médullaire, 2 muco-épidermoïdes et 1 adénocarcinome sécrétant. Dans les 6 études anatomo-pathologiques ou la différentiation était précisée, on retrouve des carcinomes peu différenciés.

Seul un patient est aujourd'hui en rémission complète, 5 sont décédés, 3 survivent avec la maladie, et 2 sont perdus de vue. Ce qui nous porte à croire que l'atteinte axillaire dans les carcinomes de la thyroïde est signe de mauvais pronostic.

Le traitement chirurgical a consisté en une thyroïdectomie totale avec curage ganglionnaire curatif de toutes les aires ganglionnaires atteintes. L'IRA-thérapie est fréquemment administrée. Elle ne l'a pas été dans 1 cas classé stade I selon la classification TNM (Tumour-Nodes-Metastases) [8]. Ce patient présenta des métastases pulmonaires 6 ans plus tard. Partant du fait que la présence d'adénopathies axillaires est de mauvais pronostic dans les cancers de la thyroïde, il serait utile de préconiser l'IRA-thérapie dans tous les cas, quelque soit la stadification. La radiothérapie et la chimiothérapie peuvent être indiqués dans les traitements palliatifs de certains types histologiques dépassés (Table 1).

**Table 1:** Traitement adjuvant en fonction du type histologique et des métastases

	Type histologique	Métastases	Traitement
1	Papillaire	Multiples	IRA thérapie
2	Muco-epidermoide	Poumons	IRA thérapie+ CTH + RTH
3	Muco-epidermoide	Poumons + Vertèbres	CTH
4	Papillaire	Absence	Absence
5	Adénocarcinome	Absence	IRA thérapie
6	Papillaire	Multiples	Non précisé
7	Médullaire	Multiples	Non précisé
8	Papillaire	Multiples	Non précisé
9	Papillaire	Poumons	IRA thérapie
10	Papillaire	Poumons + os	IRA thérapie

## Conclusion

En conclusion, bien qu'exceptionnelle, la présence de métastases axillaires d'un carcinome thyroïdien est de pronostic péjoratif. On se demande alors si ces patients ne nécessitent pas une prise en charge particulière. Une réflexion à une stratégie thérapeutique est donc nécessaire.
